# Genomic prediction of maize microphenotypes provides insights for optimizing selection and mining diversity

**DOI:** 10.1111/pbi.13420

**Published:** 2020-06-08

**Authors:** Xiaoqing Yu, Samuel Leiboff, Xianran Li, Tingting Guo, Natalie Ronning, Xiaoyu Zhang, Gary J. Muehlbauer, Marja C.P. Timmermans, Patrick S. Schnable, Michael J. Scanlon, Jianming Yu

**Affiliations:** ^1^ Department of Agronomy Iowa State University Ames IA USA; ^2^ Plant Biology Section School of Integrative Plant Science Cornell University Ithaca NY USA; ^3^ Department of Plant Biology University of Georgia Athens GA USA; ^4^ Department of Agronomy and Plant Genetics University of Minnesota St. Paul MN USA; ^5^ Center for Plant Molecular Biology University of Tübingen Tübingen Germany

**Keywords:** maize, shoot apical meristem, genetic diversity, genomic selection, genomics, plant breeding

## Abstract

Effective evaluation of millions of crop genetic stocks is an essential component of exploiting genetic diversity to achieve global food security. By leveraging genomics and data analytics, genomic prediction is a promising strategy to efficiently explore the potential of these gene banks by starting with phenotyping a small designed subset. Reliable genomic predictions have enhanced selection of many macroscopic phenotypes in plants and animals. However, the use of genomicprediction strategies for analysis of microscopic phenotypes is limited. Here, we exploited the power of genomic prediction for eight maize traits related to the shoot apical meristem (SAM), the microscopic stem cell niche that generates all the above‐ground organs of the plant. With 435 713 genomewide single‐nucleotide polymorphisms (SNPs), we predicted SAM morphology traits for 2687 diverse maize inbreds based on a model trained from 369 inbreds. An empirical validation experiment with 488 inbreds obtained a prediction accuracy of 0.37–0.57 across eight traits. In addition, we show that a significantly higher prediction accuracy was achieved by leveraging the *U* value (upper bound for reliability) that quantifies the genomic relationships of the validation set with the training set. Our findings suggest that double selection considering both prediction and reliability can be implemented in choosing selection candidates for phenotyping when exploring new diversity is desired. In this case, individuals with less extreme predicted values and moderate reliability values can be considered. Our study expands the turbocharging gene banks *via* genomic prediction from the macrophenotypes into the microphenotypic space.

## Introduction

Diverse germplasm accessions preserved at gene banks represent a critical genetic resource to tackle challenges in crop improvement and food security (Esquinas‐Alcázar, 2005; Mascher *et al*., 2019; McCouch *et al*., 2013; Varshney *et al*., 2012). Recent studies have presented genomic prediction as a cost‐effective strategy to assess and utilize potentially valuable, but poorly explored germplasm to realize the full potential of genetic resources (Crossa *et al*., 2016; Crossa *et al*., 2017; Li *et al*., 2018a; Yu *et al*., 2016). As a key enabling technology in crop improvement (Hickey *et al*., 2019), genomic selection has been rapidly incorporated in many different aspects of plant breeding (Bernardo and Yu, 2007; Crossa *et al*., 2017; Cui *et al*., 2019; Guo *et al*., 2019; Heffner *et al*., 2009; Li *et al*., 2018b). At the same time, gene bank genomics research is needed to bridge the gap between conserved genetic diversity and plant breeding (Mascher *et al*., 2019; McCouch *et al*., 2013). While genomic selection has been extensively studied for macrophenotypes such as plant height, grain yield and milk yield (Bartholomé *et al*., 2016; Crossa *et al*., 2017; Hayes *et al*., 2013; Riedelsheimer *et al*., 2012; Xu *et al*., 2014), it has not yet been extended to analyses of microscopic phenotypes, which are measured in much smaller scales.

The shoot apical meristem (SAM) comprises a population of pluripotent stem cells that generate all the above‐ground organs in plants. Although microscopic in size, SAM morphology correlates with adult plant phenotypes such as flowering time, leaf number, stem width and other agronomic traits (Leiboff *et al*., 2015; Thompson *et al*., 2015). In fact, recent studies have found that crop cultivars with improved harvest index and decreased lodging, which contributed significantly to the improved global food security during the ‘green revolution’ (Pingali, 2012), were developed with genetic materials containing natural mutations in genes involved in the SAM size regulation network (Pingali, 2012; Serrano‐Mislata *et al*., 2017). Gene circuitries acting in SAM were shown to regulate adult plant architectural traits (Knauer *et al*., 2019). Thus, it is possible that characterizing and predicting SAM morphological variation in natural populations of maize may help bridge the gap between untapped gene bank accessions and the development of elite cultivars.

In addition to improvements in genomic selection models, advances have been made in generating and leveraging other information for selecting germplasm (Crossa *et al*., 2017). For example, in a typical genomic prediction experiment, selections are often based on predicted values of a trait, such as maturity or yield. However, there are different uncertainties associated with these predicted values. Recently, the upper bound for reliability (*U*) has been proposed as an additional parameter for selection (Karaman *et al*., 2016). *U* sets the upper bound for reliability of the prediction achievable with the given training set when heritability approaches one. It is derived directly from genomewide marker data to quantify the estimability of genomic make‐up of a selection candidate from genomic data of the training set. In an earlier study, the overall *U* value difference was observed for two validation populations with different prediction accuracy values (Yu *et al*., 2016). Other studies also stressed the importance of considering other reliability estimates in optimizing the training set design (Akdemir *et al*., 2015; He *et al*., 2016; Rincent *et al*., 2017; Rincent *et al*., 2012). Although computer simulations have demonstrated the influence of reliability on prediction accuracy, this has not been explicitly demonstrated with large‐scale designed experiments. In the present study, we designed a validation set with high‐*U* values and a contrasting low‐*U* set from a diverse maize population to empirically evaluate prediction accuracy for SAM traits.

Maize is an important food crop in the world in terms of total grain production, with over one billion metric tons produced in recent years (USDA FAS, 2020). A tremendous amount of germplasm is preserved in various gene banks, representing the diversity spanning the domestication and improvement phases of maize. For instance, there are 19 780 maize accessions maintained in the USDA Plant Introduction Station at Ames, IA, USA. Adapting established genomic prediction approaches (Crossa *et al*., 2017; Meuwissen *et al*., 2001) will enable scientists to predict trait values for accessions in gene bank collections after phenotyping much smaller training sets (Crossa *et al*., 2016; Yu *et al*., 2016). Having both predicated trait values and associated reliability values available for a large number of gene bank accessions would facilitate both germplasm evaluation and utilization processes (Yu *et al*., 2016).

Here, we report a proof‐of‐concept study on the use of genomic prediction for maize SAM morphology. Established tissue processing and imaging protocols enabled us to obtain SAM morphometric measurements for a population of diverse maize inbred accessions, phenotypes that are obtainable within a much shorter time span than is required for adult phenotypes to experimentally validate genomic selection methods.

## Results

### Training population and cross‐validation

**Figure 1:**
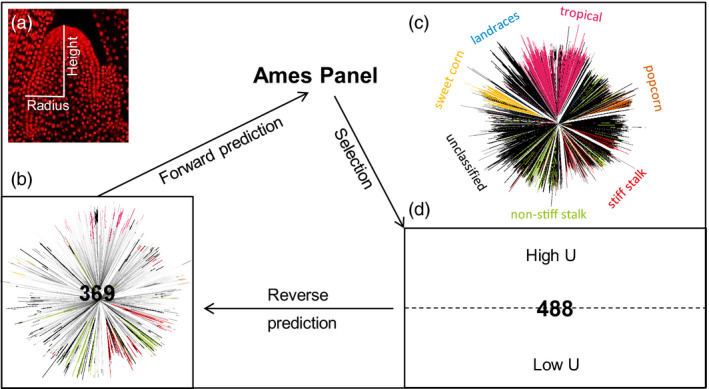
Study design of genomic prediction of maize shoot apical meristem (SAM). (a), Maize SAM contains a group of pluripotent stem cells that generate all above‐ground organs. Height and radius were two directly measured SAM traits. (b) The 369‐accession training set. A neighbour‐joining tree is presented to show the genetic diversity of this set. (c) Neighbour‐joining tree of the entire Ames Panel (*n* = 3056). The branches were colour‐coded by the associated maize groups. (d) The selected and phenotyped 488‐accession validation set. Both the 369‐accession training set and 488‐accession validation set are parts of the entire Ames Panel. Forward prediction, selection, validation and reverse prediction were performed progressively. *U* stands for upper bound for reliability. High‐*U* set has 244 accessions with high *U* values and likewise for low‐*U* set.

A set of diverse maize inbred accessions that represent maize genetic diversity (Flint‐Garcia *et al*., 2005; Liu *et al*., 2003) comprised the 369‐accession training set (Figure 1). SAM height and radius were measured traits and six additional morphometric traits were calculated, which were also used in our previous GWAS analysis of SAM diversity in maize (Leiboff *et al*., 2015). The entry mean‐based heritability for the eight traits ranged from 0.37 to 0.86 (Table S1) and was 0.82, 0.74 and 0.84 for height, radius and volume, respectively. SAM height had a mean of 117 µm and a standard deviation of 19 µm, and radius had a mean of 76 µm and a standard deviation of 6 µm (Leiboff *et al*., 2015). Correlation between SAM height and radius was 0.58, and their correlations with volume were 0.85 and 0.89, respectively. Among five other calculated traits, the correlation for arch length with SAM height and radius was 0.98 and 0.72, respectively, and for parabolic coefficient, it was 0.35 and −0.50, respectively.

**Figure 2:**
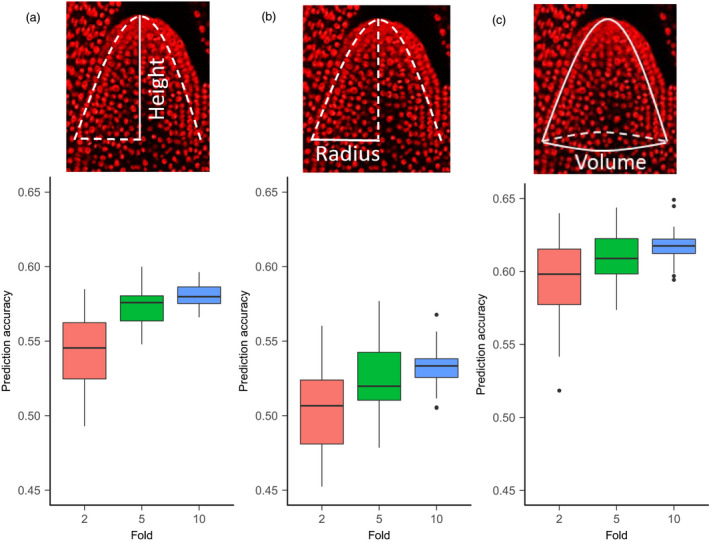
Prediction accuracy in the 369‐accession training set through cross‐validation. (a) Graphical illustrations of three SAM traits: height, radius and volume. Height (*h*) and radius (*r*) are directly measured under microscope; volume is calculated through a parabolic model. (b) Prediction accuracy is estimated through two‐, five‐ and 10‐fold cross‐validation.

We first conducted the standard *k*‐fold cross‐validation to assess prediction accuracy using the SAM phenotypes and the genomewide SNP data (*n* = 435 713 SNPs) for the 369‐accession training set. We found that the twofold, fivefold and 10‐fold cross‐validation gave relatively stable prediction accuracy for all eight traits. The 10‐fold cross‐validation gave prediction accuracy values of 0.58, 0.53 and 0.62 for height, radius and volume, respectively (Figure 2), with an overall range from 0.13 to 0.63 (Table S1). Given the generally moderate to‐high trait heritability, this level of prediction accuracy was expected. For further analyses, this entire 369‐accession set was used as the training population to establish the prediction models for all traits.

### Genomic prediction and validation set design

**Figure 3:**
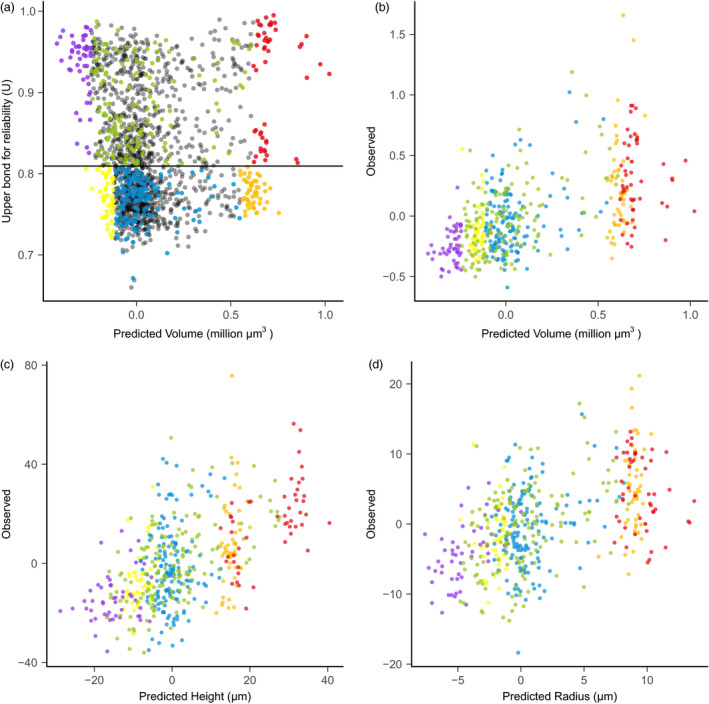
Validation set selection and empirical validation of shoot apical meristem (SAM) volume. (a) The validation set was built with six parts indicated by different colours: (i) 50 accessions with the large predicted SAM volume and high *U* values (red); (ii) 50 accessions with the small SAM volume and high *U* values (violet); (iii) 150 random accessions from the remaining accessions with high *U* values (green); (iv) 50 accessions with the large SAM volume and low *U* values (orange); (v) 50 accessions with the small SAM volume and low *U* values (yellow); and (vi) 150 random accessions from the remaining accessions with low *U* values (blue). Not selected accessions are shown in grey. (b) Prediction accuracy for volume. (c) Prediction accuracy for height. (d) Prediction accuracy for radius. The accuracy was calculated from the 488‐accession set that were successfully phenotyped.

The average *U* value was 0.82 for the 2687‐accession selection candidate set (Figure 3a), which indicates overall close genomic relationship between the 369‐accessoin training set and the selection candidate set. As *U* values decrease, the predicted values for SAM volume become less extreme. Six combinations of *U* values and predicted volume values allowed us to assess the effects of *U* values on prediction accuracy (Figure 3a).

### SAM phenotyping for empirical validation

Using the same phenotyping protocols as described in the previous study (Leiboff *et al*., 2015), we obtained measures of SAM height and SAM radius for 488 accessions (out of the originally planned 500 accessions), termed as the 488‐accession validation set. SAM height ranged from 50 µm ~ 200 µm and SAM radius from 40 µm ~ 110 µm, indicating a wide range of variation. Six other SAM traits were derived using the parabolic model from the height and radius measurements. Distributions of height, radius and volume (Figure S1) are consistent with the original training set (Leiboff *et al*., 2015). Entry mean‐based heritability was 0.70, 0.59 and 0.65 for height, radius and volume, respectively (Table S2).

Prediction accuracy for the entire 488‐accession validation set (correlation between the predicted values and experimentally observed trait values) was 0.56, 0.57 and 0.51 for volume, height and radius, respectively (Figure 3b‐d). Prediction accuracy across eight traits varied from 0.37 (parabolic coefficient) to 0.57 (height, area and arc length) (Table S2), and these prediction accuracy values were close to those from the cross‐validation (Figure 2, Table S1). Adjusted prediction accuracy ranged from 0.47 (parabolic coefficient) to 0.69 (Volume) (Table S2). Because the square of SAM radius is used in the calculation of volume, radius influenced the predicted value of volume more than height. As a result, the distribution pattern of the observed *versus* predicted values were similar between volume and radius (Figure 3b‐d); this was verified by checking a sample of five accessions with different predicted values for volume and radius (Figure S2). Although the overall observed values for six designed sets were as predicted, some accessions in the low‐*U* set had either very large or very small observed trait values (Figure 3).

### Prediction accuracy for high‐*U* and low‐*U* sets

**Figure 4:**
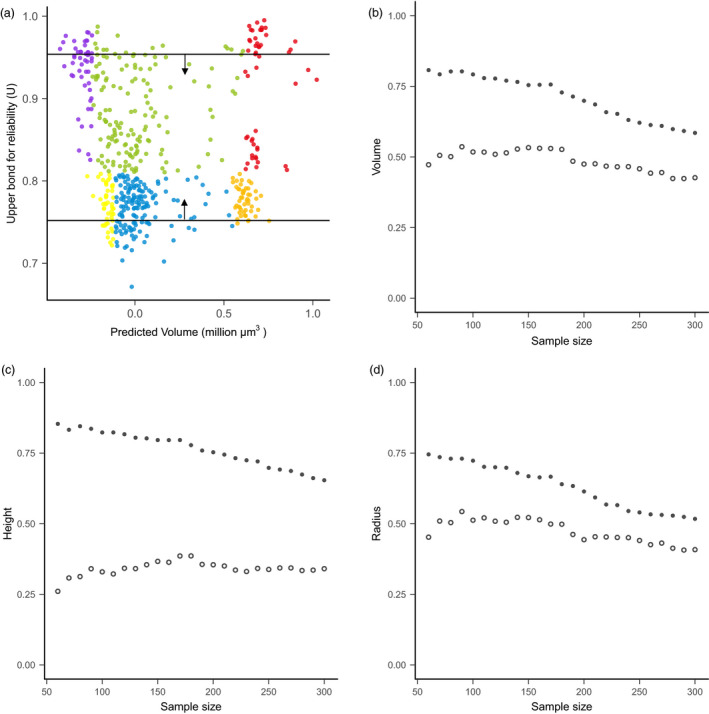
Prediction accuracy comparison by sampling accessions with different *U* values. (a) Graphical illustration of the sampling process. The sampling was performed along the *y*‐axis according to *U* value. Sample size ranges from 60 to 300 with increment of 10. For example, when *n* = 60, a contrast of two sets of materials was formed, including the high‐*U* set with the top 60 and the low‐*U* set with the bottom 60 dots. (b–d) Comparison of prediction accuracy between the high‐*U* set (solid circle) and low‐*U* set (open circle) for volume (b), height (c), and radius (d). Notice that for the high‐*U* set, increasing the sample size of the validation set leads to the inclusion of individuals with gradually smaller *U* values, thus reduced prediction accuracy.

The 244‐accession set with high‐*U* values showed significantly higher prediction accuracy than the low‐*U* set for all SAM traits (Figure S3, Table S3). For instance, prediction accuracy for volume is 0.64 for the high‐*U* set, but 0.46 for the low‐*U* set. The difference between high‐*U* and low‐*U* sets is more pronounced for traits with high heritability and when the validation sets with a smaller sample size were compared (Table S3). Gradient sampling analysis from both ends of *U* values indicated that the high‐*U* sets consistently displayed better prediction accuracy (Figure 4, Figure S4). On average, the high‐*U* lines showed an accuracy advantage of +0.22, +0.42 and +0.16 for volume, height and radius, respectively. This accuracy advantage was more pronounced for the smaller sample size (larger contrast in average *U* between two groups) than the larger sample size (smaller contrast in average *U*) (Figure 4).

### Different maize groups and their associated SAM volumes

**Figure 5:**
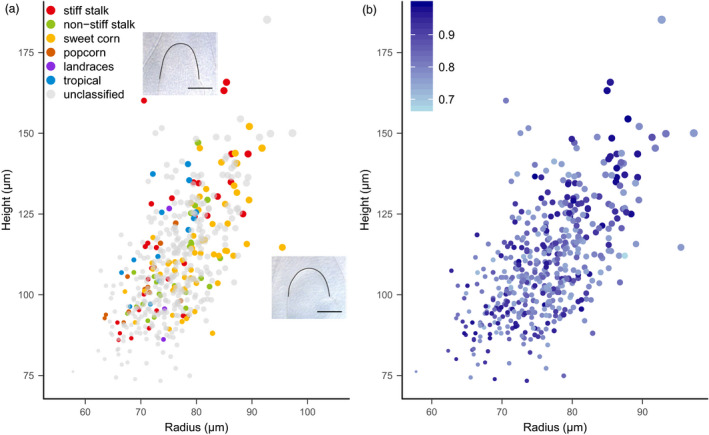
Relationship among shoot apical meristem (SAM) traits, maize groups and upper bound for reliability (*U*). (a) SAM height and radius in the 488‐accession validation set. Dot size indicates SAM volume; dot colour indicates maize groups. The two inserts are two images of SAMs with different height and radius. Scale bar, 100 µm. (b) Different maize accessions have different *U* values. Colour intensity indicates the *U* value.

Interestingly, we noticed that accessions with large predicted SAM volume values were located apart from other accessions (Figure 3b). We performed further investigation on the biological backgrounds of these accessions (Figure 5). In general, different maize groups show overlapping SAM shape and volume (Figure 5a). We then found that, within the relevant ranges of trait values, the distance of individual accessions to the origin of the *x*‐*y* (radius‐height) plot approximates the SAM volume (Figure S5). This indicates that the accessions in the upper right region of the *x*‐*y* plot have large SAMs, while accessions in the lower left region have small SAMs. Tropical accessions and popcorns have relatively smaller SAMs, while large SAMs predominate in sweet corn and stiff stalk accessions (Figure 5a).

As illustrated by the two SAM images, the stiff stalk accessions had relatively taller SAMs (large height) and the sweet corn accessions had relatively wider SAMs (large radius) than other accessions. The stiff stalk group had higher *U* values (Figure 5b), which was due to the high proportion of stiff stalk accessions in the 369‐accession training set. In contrast, a smaller proportion of sweet corn accessions were included in the training set, resulting in generally lower *U* values for the sweet corn accessions (Figure S6).

### Reverse prediction

To further demonstrate the effect of genomic relationship between training set and validation set on prediction accuracy, we conducted a reverse prediction. As expected, the 244‐accession high‐*U* set outperformed the 244‐accession low‐*U* set when it was used to predict SAM traits of the original 369‐accession set (Table 1, Table S4). For example, prediction accuracy for height was 0.61 when using the high‐*U* set, but only 0.21 when using the low‐*U* set to build the reverse prediction model. On average, using the high‐*U* set to build the model yielded an average prediction accuracy 113% higher than using the low‐*U* set across eight SAM traits (Table 1, Table S4).

**Table 1:** Comparison of prediction accuracy for forward and reverse prediction

Trait	Forward prediction	Reverse prediction
	T369 → V488	HighU244 → T369	LowU244 → T369
Height	0.57	0.61	0.21
Radius	0.51	0.53	0.30
Volume	0.56	0.62	0.34

The forward prediction uses the 369‐accession training set (T369) to train the model and predict the 488‐accession validation set (V488). The reverse prediction uses the subsets of the V488 (244‐accession high‐*U* set or 244‐accession low‐*U* set) to train the model and predict the T369. *U*, upper bound for reliability.

## Discussion

### Genomic prediction of microscopic traits

In the earlier study, morphometric diversity across 369 maize inbred lines was examined by modelling the SAM shape as a parabola (Leiboff *et al*., 2015). With 435 713 genomewide SNPs, we first obtained the genomic prediction models for SAM traits. We then generated the predicted SAM trait values for 2687 untested accessions and conducted the phenotyping experiment to empirically validate the predictions with 488 accessions. Therefore, this study expands the power of genomic prediction into microscopic phenotypes. The obtained prediction accuracy values are encouraging for two reasons. First, a diverse genetic context represented by the maize Ames Panel was examined. Second, this study represents the first attempt to obtain robust phenotypic data and predictions for microscopic traits. Similar to adult plant traits (Crossa *et al*., 2017), increased prediction accuracy can be achieved with improved prediction models, better designed training sets and more robust phenotype for the training set from enhanced phenotyping methods and increased replications. In the current study, four replications were used in phenotyping the training set and three for the validation set. An increase in the number of runs, replications per run and plants per replication would enhance the prediction. On the other hand, the obvious limitation of this study is the lack of using different environments (year by location combinations), which is necessary for general quantitative traits with agronomic importance in plants. Fortunately, significant progress has been made for genomic prediction under genotype‐by‐environment interaction (Crossa *et al*., 2017), and an integrated framework to capture the major patterns has been demonstrated (Guo *et al*., 2020; Li *et al*., 2018b).

This microscopic phenotype prediction could also provide new insights into how genomic prediction in the gene banks can be leveraged from a biological perspective. For example, studies can be designed to investigate any contrasting adult phenotypes between accessions with predicted large *versus* small SAMs (*i.e*. volume) or between extreme SAM height or radius values. These comparisons can also be conducted for different maize groups. Our results also indicate that if more representative materials from a subgroup are available in the training set, researchers can expect an improved prediction accuracy for the related materials in the validation set. These findings agree with the general understanding of genomic selection principles and empirical findings for agronomic traits (de los Campos *et al*., 2010; Crossa *et al*., 2017).

Previous work (Leiboff *et al*., 2015) identified certain correlations between some SAM‐related traits and agronomically important adult traits such as flowering time, stem size and leaf node number, which leads us to speculate that it is possible to infer similar results for key traits for breeding. Another recent study also indicated the shared gene circuitries acting in SAM and adult plant architectural traits (Knauer *et al*., 2019). However, additional research is needed to examine these relationships, particularly if field experiments can be carried out to allow both the destructive tissue sampling for SAM measurements as well as the measuring of adult plant traits with the remaining plants in the plot.

### Genomic selection considering both prediction and reliability

In classical breeding, individuals are selected by considering both the means and the accuracies of the trials. In molecular breeding, consideration of reliability on top of the prediction value has also been proposed in animal breeding (Hayes *et al*., 2009; VanRaden *et al*., 2009). Rincent *et al*. (2012) and Akdemir *et al*. (2015) applied reliability to optimize the training population according to the genetic composition of selection candidates. In addition, He *et al*. (2016) found genomic prediction accuracy was substantially higher in subpopulations with higher reliability. Rincent *et al*. (2017) further proposed that training set design should consider both performance predictions and the reliability of these predictions. Nevertheless, cross validation was used in these studies and the benefit of considering reliability in designing genomic selection has not been tested with actual validation experiments.

The *U* value (Karaman *et al*., 2016) contributes three desirable features to this task: (i) *U* is derived directly from the marker data and it doesn’t require any phenotype information; (ii) its calculation is independent of trait heritability; and (iii) the *U* value ranges from 0 to 1, wherein higher *U* values render more reliable predictions. Accordingly, interpretation of findings is simplified across different populations and traits. Although these features were noticed earlier, *U* values were only calculated afterwards to explain the findings, rather than explicitly considered in the experiment design (Yu *et al*., 2016). In the present study, we designed two contrasting groups of high‐*U* set and low‐*U* set with different SAM volume predictions and empirically examined the prediction accuracy difference for SAM volume and seven other traits. Checking genetic backgrounds of the validation set and the training set and conducting reverse prediction generated relevant findings.

Our overall findings provide support to the ‘double selection’ (prediction & reliability) concept to enhance genomic selection. Once the predicted trait values and reliability values are obtained for all selection candidates, how to select individuals for further phenotyping can have two options. In cases where the focus is to identify untested individuals with the best potential, selecting individuals with extreme predicted values should be advantageous, even though many selection candidates with extreme predicted values would generally have high‐*U* values. On the other hand, if exploring additional genetic diversity different from what is captured in the initial training set is also desirable, individuals with moderate *U* values but less extreme predicted values may be considered. This is particularly the case when a large genetic space of a gene bank collection is being explored through an iterative model building and updating process with different cycles of phenotyping–predicting–phenotyping (Yu *et al*., 2016).

Further research can be conducted to investigate whether *U* value can be used to optimize training set design and whether it can outcompete various other parameters (Akdemir *et al*., 2015; Guo *et al*., 2019; He *et al*., 2016; Rincent *et al*., 2017; Rincent *et al*., 2012) and be incorporated into optimal design methods from data mining (Guo *et al*., 2019). This is highly relevant when the genotyping information of the entire population is available at the beginning of the genomic selection project, which will be the case for genomics‐of‐gene bank research (Mascher *et al*., 2019; Yu *et al*., 2016) and is the case for hybrid prediction from fixed sets of inbreds (Guo *et al*., 2019). Conducting this type of studies with complex agronomic traits would also be highly desirable.

### Turbocharging gene banks for crop improvement

Genetic resources are critically important for food security (Mascher *et al*., 2019; McCouch *et al*., 2013). Our results demonstrate that microscopic phenotypes can be predicted through a genomewide prediction model to help germplasm evaluation and selection. The prognostic value of SAM morphology to adult plant phenotypes can be further studied to leverage these predictions. More importantly, by utilizing a trait that can be rapidly measured, we experimentally demonstrated the differences in prediction accuracy for different sets of validation materials. Through double selection considering both predicted value and reliability of the prediction, breeders can make informed decision to leverage the efficiency of genomic selection programmes and explore the large genetic space.

## Experimental procedures

### Study design and key populations

The goal of this study is to explore the general approach in germplasm enhancement and genomic selection. We provide here a brief summary of the overall experimental design: (i) establishing the prediction model with a diverse training set; (ii) making genomic predictions (*i.e*. forward prediction) and obtaining the *U* values for the untested accessions; (iii) choosing a validation set with either high‐*U* or low‐*U* accessions and phenotyping this validation set; (iv) assessing prediction accuracy as a whole and separately for the high‐*U* and low‐*U* sets; and (v) conducting additional analyses by generating reverse prediction using the high‐*U* or low‐*U* set separately as the training sets.

Two populations used in this study include the training population and selection candidate population. The set of 369 diverse maize inbred accessions included most of the maize diversity panel (Flint‐Garcia *et al*., 2005; Liu *et al*., 2003) and additional maize inbreds with expired Plant Variety Protect Act certification (Leiboff *et al*., 2015). The selection candidate population is the 2687‐accession set, which is the other part of the entire 3056‐accession Ames Panel (Romay *et al*., 2013).

### Phenotyping of the training population

Phenotypic data of SAM traits and genotypic data for this 369‐accession set detailed in our previous publication (Leiboff *et al*., 2015) were used as the starting information for the current study (Data S1). Established procedures of plant growth, SAM tissue preparation and image processing were followed (Leiboff *et al*., 2015). Briefly, plants were grown under standard conditions with 10‐hr day cycles in Percival A100 growth chambers (Percival Scientific, Perry, IA) planted in 96‐well trays with all edge positions filled with inbred B73. Soil media was a 1:1 mixture of Turface MVP (PROFILE Products LLC, Buffalo Grove, IL) and LM111 (Lambert Peat Moss, QC, Canada). All plants were harvested 14 days after planting and quickly trimmed to small SAM‐containing tissue cassettes and fixed overnight in FAA (3.7% formalin, 5% acetic acid and 50% ethanol in water) on ice. After dehydration and clearing in methyl salicylate, single optical sections of SAM tissue were selected at near‐median longitudinal planes for image analysis, using a high‐throughput approach including custom ImageJ macros and Python scripts to process images. SAM height (*h*) and radius (*r*) were directly measured from the 2D SAM image, and the remaining six traits were calculated using an approximation of the SAM shape with a parabolic model (Leiboff *et al*., 2015). The formulas are as follows: height/radius = $\frac{h}{r}$, area = $\frac{4}{3}\left(\mathrm{hr}\right)$, volume = $\frac{\pi }{2}{\mathrm{hr}}^{2}$, parabolic coefficient $a=\frac{h}{r^{2}}$, surface area = $\frac{\pi r}{6h^{2}}\left[{\left(r^{2}+4h^{2}\right)}^{\frac{3}{2}}-r^{3}\right]$, and arc length = $\sqrt{r^{2}+4h^{2}}+\left(\frac{r^{2}}{2h}\right)\sin\;h^{-1}\left(\frac{2h}{r}\right)$. Four replications were used in the phenotyping experiment, and variance component analysis was conducted to obtain the entry‐mean‐based heritability (*h^2^*).

### Genotyping data processing

The Ames Panel, including a 369‐accession training set and 2687 selection candidates, were genotyped with genotyping by sequencing (GBS) (Romay *et al*., 2013). Briefly, leaf samples were collected for DNA extraction and *Ape*KI restriction enzyme was used for library generation. Multiplexed 96 samples were sequenced by each Illumina flow cell lane. Sequence data were processed through the GBS SNP discovery pipeline in TASSEL (Bradbury *et al*., 2007). The pipeline parameters included minimum SNP call rate of 10%, minimum inbreeding coefficient of 0.8, and MAF of 0.2%. The GBS process produced 681 257 SNPs distributed across the entire genome. After the 369‐accession training set and 2687 selection candidates in the current study were established, we removed monomorphic SNPs and extracted 435 713 common SNPs shared between the 369‐accession training set and the 2687 selection candidates for genomic prediction analysis.

### Genetics analysis

Principal component analysis was conducted in GAPIT (Lipka *et al*., 2012), and the results were visualized in R. Neighbour‐joining trees were generated in TASSEL (Bradbury *et al*., 2007), and the result was then visualized in the R package ‘ape’ (Popescu *et al*., 2012). To reduce the computational burden, 1% of SNPs (*n* = 4358) were sequentially sampled (1 out of every 100) from the original SNP list for tree construction. Group assignment for all maize accessions (stiff stalk, nonstiff stalk, tropical, landraces, popcorn, sweet corn and unclassified) was based on the earlier study (Romay *et al*., 2013).

### Genomic prediction

The 435 713 SNPs and the 369‐accession training set phenotype data were used for genomic prediction in the R package rrBLUP (Endelman, 2011) for the three key traits of interest (height, radius and volume). Prediction accuracy for cross‐validation and empirical validation was based on correlation between predicted genotypic effects (PGE) and observed phenotype (*y*), (*r_(PGE, y)_*). Adjusted prediction accuracy was calculated as prediction accuracy divided by the square root of heritability. Using three key traits, we verified the sampling of SNPs did not affect the prediction once the total SNP number is high. Accordingly, a randomly selected subset of SNPs (43 571) were used for five other traits to reduce the computation time.

In cross‐validation, we applied the *k*‐folds (*k* = 2, 5, or 10) scheme to evaluate within the 369‐accession training set. Briefly, in each run, data were randomly partitioned into *k*‐fold; predicted values for each fold were obtained with data from all other folds; after obtaining the predicted values for all folds, correlation between predicted values and observed values was calculated as the prediction accuracy. Prediction accuracy values were then averaged across 50 runs. When building the prediction model to predict the phenotype of the selection candidates, the phenotype data and SNP data for the 369‐accession training set were used to obtain the marker effects for the SNPs. These estimated marker effects were combined with the marker matrix of the selection candidates to obtain PGEs for the 2687 selection candidates (Data S2).

Upper bound for reliability was obtained for the 2687 selection candidates: $U={\hat{v}}^{\prime }\hat{v}/v^{\prime }v$, where $\hat{v}=M^{\prime }{\left(MM^{\prime }\right)}^{-1}Mv$; *M* is a matrix of training set genotypes; and *v* is a vector of validation genotype (Karaman *et al*., 2016).

### Validation design and additional SAM phenotyping

By removing the accessions with missing SNP calling rate >80% and flowering time >90 days at latitude 40°, we first reduced the selection candidates to 1711 accessions for the next step of selection. Based on predicted SAM volume and the *U* values, we selected 500 accessions as the validation set as follows: (i) 50 accessions with largest SAM volume in the high‐*U* half of the selection candidate set; (ii) 50 accessions with the smallest SAM volume in the high‐*U* half; (iii) 150 random accessions from the remaining accessions in the high‐*U* half; (iv) 50 accessions with the largest SAM volume in the low‐*U* half; (v) 50 accessions with the smallest SAM volume in the low‐*U* half; and (vi) 150 random accessions from the remaining accessions in the low‐*U* half.

These 500 accessions were grown with three replications in a greenhouse and phenotyped for SAM measurements following the same protocol used for the training set. We obtained eight SAM traits for 488 accessions and termed it as the 488‐accession validation set (Data S3). Combining data from three replications, a single set of best linear unbiased predictions (Data S4) was calculated for the 488‐accession validation set to be compared with their PGEs (Data S5). Prediction accuracy was computed for the 488‐set as a whole and separately for different subsets.

To probe the general pattern of *U* effect on prediction accuracy, we further tested a gradient of sample sizes with different *U* values. These analyses started with two contrasting groups of 60 accessions with either highest *U* values or lowest *U* values. With an increment of 10 accessions, we sequentially added samples based on their *U* values until the size reached 300. Prediction accuracy was computed for each validation set with different sizes.

### Reverse genomic prediction

Once the SAM phenotypic data were obtained for the validation set, it was possible to investigate reverse genomic prediction. In other words, we can ask whether genotyping and phenotyping data obtained for the validation data can be used to build a model to predict the phenotypes of the original training set. In this case, we used either the 244‐accession high‐*U* set or the 244‐accession low‐*U* set from the 488‐accession validation set to build the genomic prediction model. The genotype data used were the set of 43 571 SNPs. This model was then combined with the genotype data of the original 369‐accession set to generation the predicted SAM trait values, which were then correlated with the observed value to obtain the prediction accuracy for reverse prediction. This analysis provided additional results to understand the value of considering *U* in genomic prediction.

SAM phenotyping data and predicted trait data are included in supporting data (Data [Link], [Link], [Link], [Link], [Link]).

## Conflict of interest

The authors declare no conflict of interest.

## Author contributions

JY, PSS, XZ, GJM, MCPT and MJG designed the experiment; XY, SL, XL, TG and NR performed the experiment and analysed the data; and XY, SL, MJG and JY wrote the manuscript with input from other authors.

## Supporting information


**Figure S1** Phenotype distributions of the 488‐accession validation set.
**Figure S2** Shoot apical meristem (SAM) radius and volume distribution patterns.
**Figure S3** Influence of upper bound for reliability (*U*) on prediction accuracy for volume, height, and radius (*n* = 244 in each panel).
**Figure S4** Prediction accuracy comparison by sampling accessions with different *U* values for five SAM traits.
**Figure S5** Approximation of shoot apical meristem (SAM) volume by the position of the dot on the *x*‐*y* (radius‐height) plot.
**Figure S6** Relationship between the 369‐accession training set, the 488‐accession validation set, and the entire Ames Panel (*n* = 3056) revealed by principal component analysis (PCA).
**Table S1** Heritability and prediction accuracy for the 369‐accession training set.
**Table S2** Heritability and prediction accuracy for the 488‐accession validation set.
**Table S3** *U* effects on traits with different heritability.
**Table S4** Comparison of prediction accuracy for forward and reverse prediction for all traits.

**Data S1** SAM trait BLUPs for the 369‐accession training set.

**Data S2** Predicted genetic effects and *U* values for the entire selection candidates of 2687 maize accessions.

**Data S3** SAM trait values for the 488‐accession validation set.

**Data S4** Summarized SAM trait values for the 488‐accession validation set.

**Data S5** Predicted genetic effects and *U* values for the 488‐accession validation set, which is part of the 2687‐accession selection candidate set.
