# Supplementary material for: Genomic prediction of maize microphenotypes provides insights for optimizing selection and mining diversity
Source: Plant Biotechnol J. 2020 Jun 8;18(12):2456–65. doi: 10.1111/pbi.13420 (PMC7680549; doi:10.1111/pbi.13420)
Supplement: Supplementary file 1 — Figure S1 Phenotype distributions of the 488‐accession validation set. Figure S2 Shoot apical meristem (SAM) radius and volume distribution patterns. Figure S3 Influence of upper bound for reliability (U) on prediction accuracy for volume, height, and radius (n = 244 in each panel). Figure S4 Prediction accuracy comparison by sampling accessions with different U values for five SAM traits. Figure S5 Approximation of shoot apical meristem (SAM) volume by the position of the dot on the x‐y (radius‐height) plot. Figure S6 Relationship between the 369‐accession training set, the 488‐accession validation set, and the entire Ames Panel (n = 3056) revealed by principal component analysis (PCA). Table S1 Heritability and prediction accuracy for the 369‐accession training set. Table S2 Heritability and prediction accuracy for the 488‐accession validation set. Table S3 U effects on traits with different heritability. Table S4 Comparison of prediction accuracy for forward and reverse prediction for all traits. [file PBI-18-2456-s003.pdf]

Genomic prediction of maize microphenotypes provides insights for optimizing selection and mining diversity

Xiaoqing Yu[1], Samuel Leiboff[2], Xianran Li[1], Tingting Guo[1], Natalie Ronning[2], Xiaoyu Zhang[3], Gary J. Muehlbauer[4], Marja C. P. Timmermans[5], Patrick S. Schnable[1], Michael J. Scanlon[2] & Jianming Yu[1,*]

[1] Department of Agronomy, Iowa State University, Ames, IA, USA
[2] Plant Biology Section, School of Integrative Plant Science, Cornell University, Ithaca, NY, USA
[3] Department of Plant Biology, University of Georgia, Athens, GA, USA
[4] Department of Agronomy and Plant Genetics, University of Minnesota, St. Paul, MN, USA
[5] Center for Plant Molecular Biology, University of Tübingen, Tübingen, Germany

Correspondence (Tel 1 515-294-2757; fax 1 515-294-3163; email jmyu@iastate.edu)

Keywords:
Maize | shoot apical meristem | genetic diversity | genomic selection | genomics | plant breeding

Supporting Information

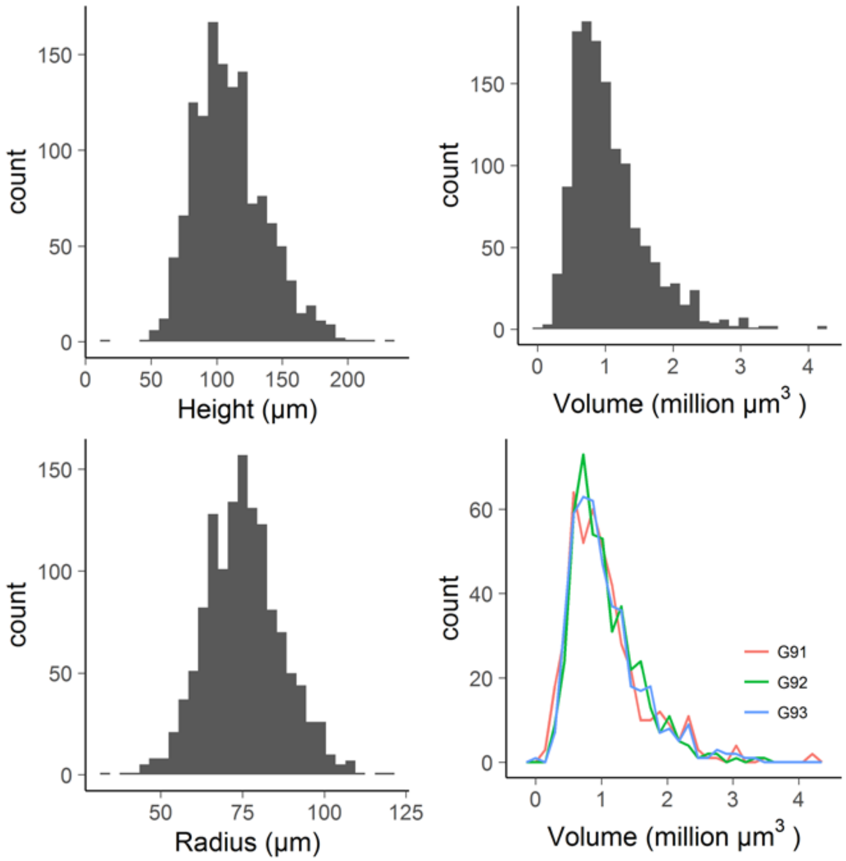

Figure S1. Phenotype distributions of the 488-accession validation set. The lower right panel shows the histograms of shoot apical meristem (SAM) volume measurements from three different replications (G91, G92, and G93).

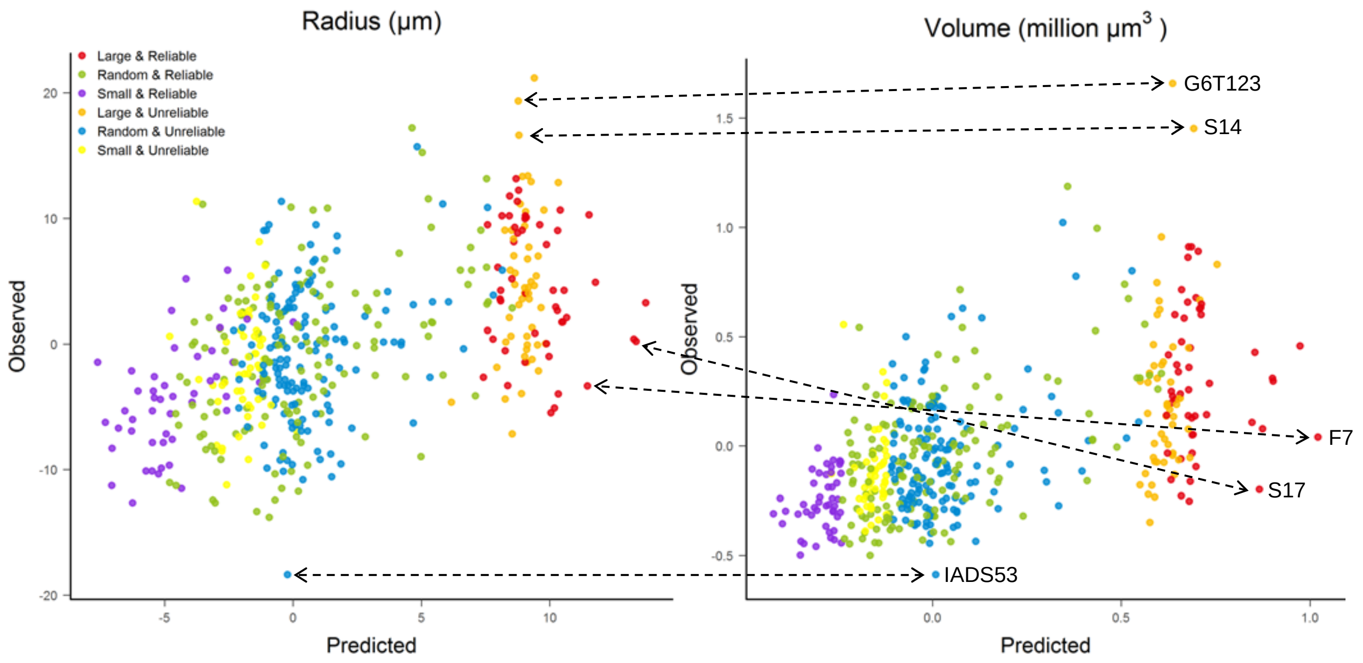

Figure S2. Shoot apical meristem (SAM) radius and volume distribution patterns. Different groups of maize accessions were selected for validation based on the predicted volume (large, small, or random) and *U* values (reliable and unreliable). Five maize accessions were marked to show the similar pattern between radius and volume.

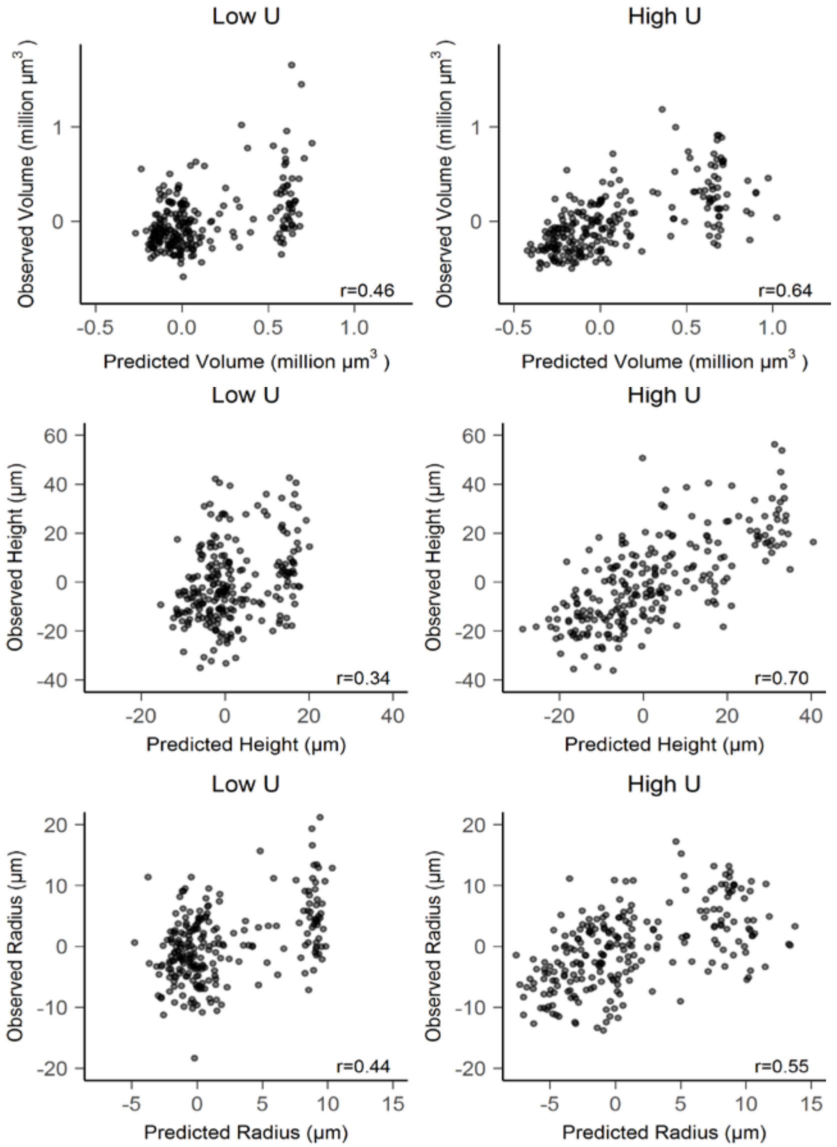

Figure S3. Influence of upper bound for reliability (*U*) on prediction accuracy for volume, height, and radius (*n* = 244 in each panel).

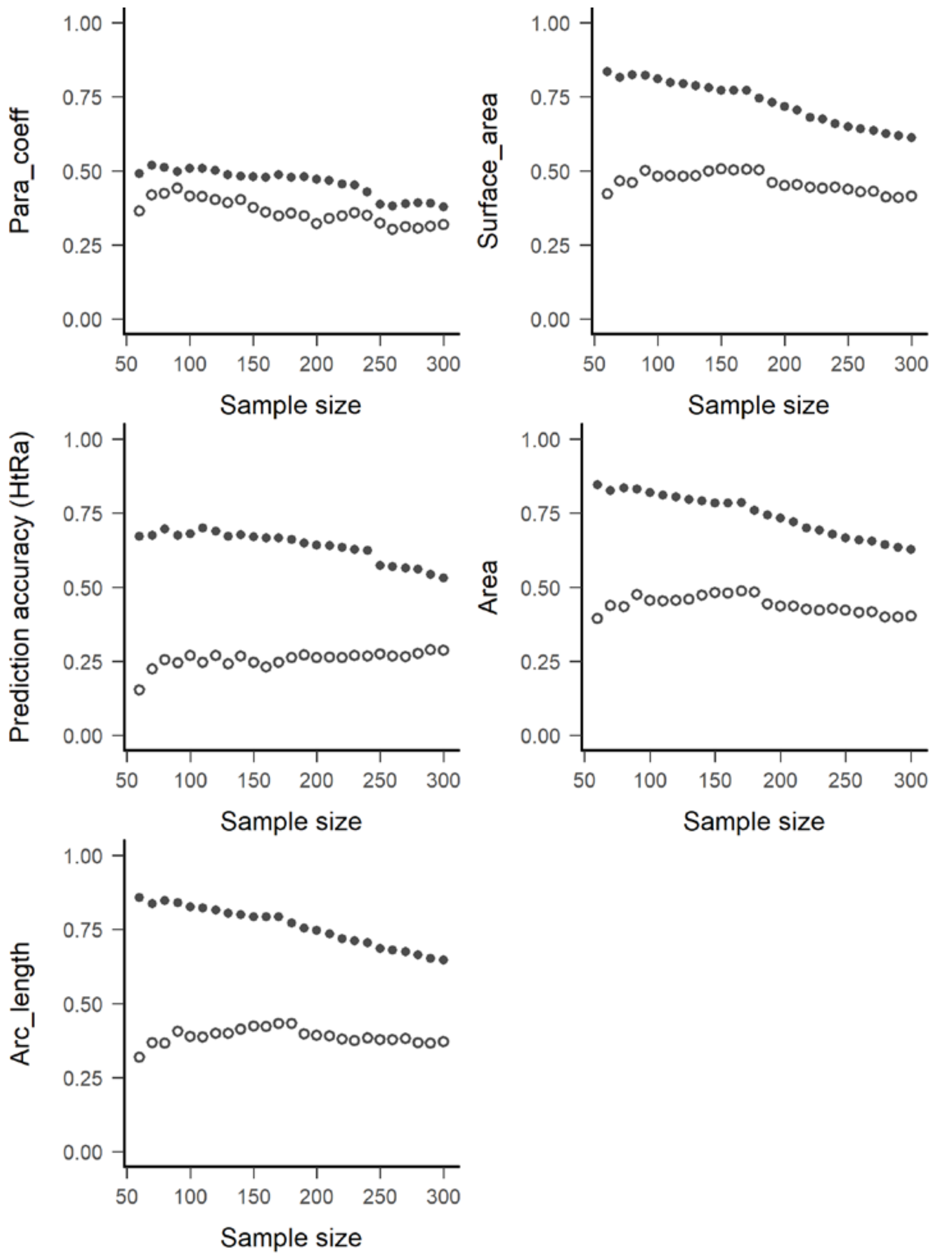

Figure S4. Prediction accuracy comparison by sampling accessions with different *U* values for five SAM traits. Each panel represents prediction accuracy comparison between the high-*U* set (solid circle) and low-*U* set (open circle) for one trait. Details of the phenotyping method can be found in the materials and methods section. Notice that for the high-*U* set, increasing the sample size of the validation set results in including individuals with gradually smaller *U* values, thus reduced prediction accuracy.

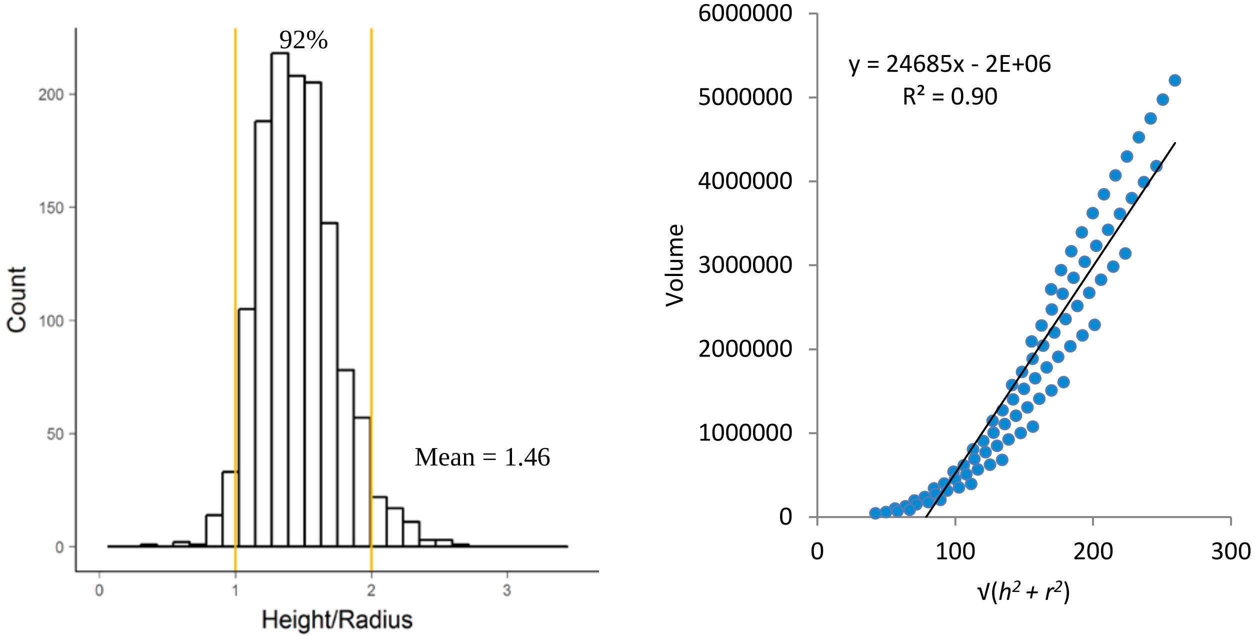

Figure S5. **Approximation of shoot apical meristem (SAM) volume by the position of the dot on the *x*-*y* (radius-height) plot.** Left panel shows histogram of the height/radius ratio of the SAM from 1,711 maize accessions. 92% of the accessions have a height/radius ratio between 1 and 2 (mean=1.46). Right panel shows the close approximation of the SAM volume by the distance of a dot to the origin of the height-versus-radius plot, with simulated data having a height/radius ratio between 1 to 2. The y-axis is SAM volume ($\frac{\pi}{2}hr^2$); the x-axis is $\sqrt{h^2+r^2}$ (the distance from the dot to the origin in **Fig. 5**).

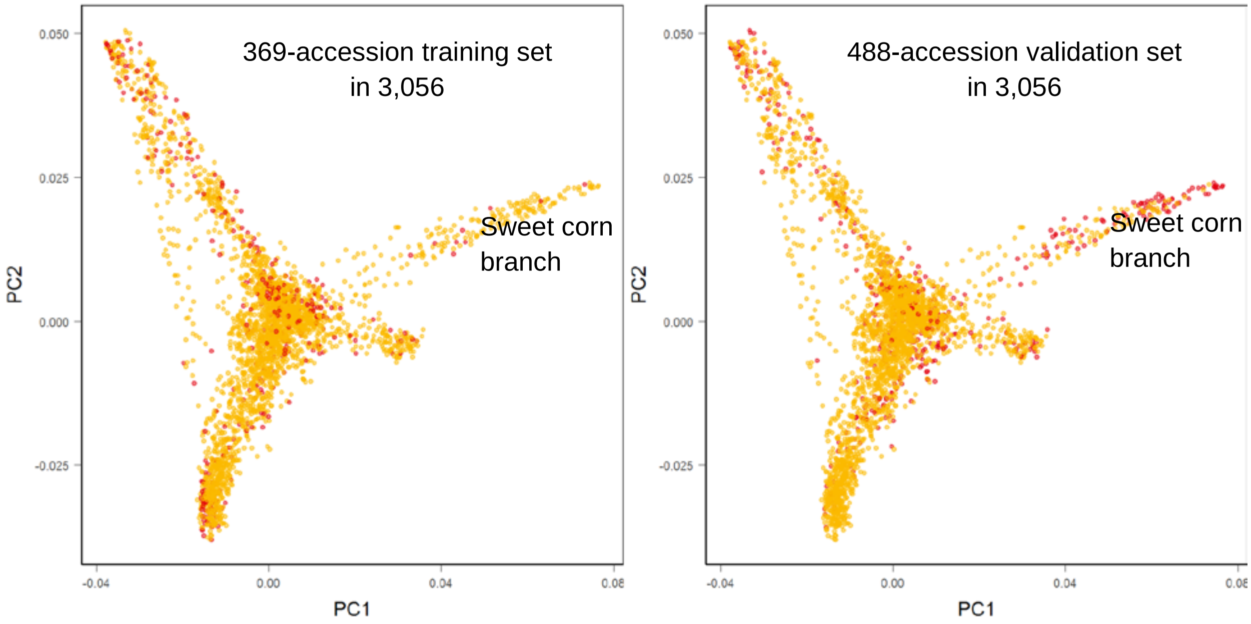

Figure S6. **Relationship between the 369-accession training set, the 488-accession validation set, and the entire Ames panel (*n* = 3,056) revealed by principal component analysis (PCA)**. The 369-accession training set is indicated by red dots in the left panel; the 488-accession validation set is indicated by red dots in the right panel; other accessions in the Ames Panel are indicated by orange dots.

Table S1. Heritability and prediction accuracy for the 369-accession training set. h^2, entry-mean based heritability, was obtained from 4 replications. Prediction accuracy was estimated from 10-fold cross-validation with 50 iterations.

Trait	h^2	h	Prediction Accuracy (Cross Validation)
Parabolic coefficient	0.37	0.61	0.13
Height/Radius	0.58	0.76	0.32
Radius (µm)	0.74	0.86	0.53
Height (µm)	0.82	0.91	0.58
Volume (million $µm^3$)	0.84	0.92	0.62
Surface area ($µm^2$)	0.86	0.93	0.63
Area ($µm^2$)	0.86	0.93	0.63
Arc length (µm)	0.86	0.93	0.62
Average across 8 traits	0.74	0.86	0.51

Table S2. Heritability and prediction accuracy for the 488-accession validation set. h^2, entry-mean based heritability, was obtained from 3 replications. Prediction accuracy was calculated as the correlation between the predicted trait values and experimentally observed trait values. Adjusted prediction accuracy was calculated using prediction accuracy divided by the square root of heritability (h).

Trait	h^2	h	Prediction Accuracy (Empirical Validation)	Adjusted Prediction Accuracy (Empirical Validation)
Parabolic coefficient	0.63	0.79	0.37	0.47
Height/Radius	0.73	0.85	0.45	0.53
Radius (µm)	0.59	0.77	0.51	0.66
Height (µm)	0.70	0.84	0.57	0.68
Volume (million $µm^3$)	0.65	0.81	0.56	0.69
Surface area ($µm^2$)	0.75	0.87	0.56	0.64
Area ($µm^2$)	0.76	0.87	0.57	0.66
Arc length (µm)	0.77	0.88	0.57	0.65
Average across 8 traits	0.70	0.84	0.52	0.62

Table S3. *U* effects on traits with different heritability. Prediction accuracy was assessed using the subsets of the validation set (488) with the sorted *U* values. *U* values generally have a greater effect for traits with high heritability. The list of traits is sorted by their heritability.

Trait	h^2 (training)	1/2 Validation Set (n_1=n_2=244)			1/4 Validation Set (n_1=n_2=122)			~1/10 Validation Set (n_1=n_2=50)		
		Low *U*	High *U*	Diff	Low *U*	High *U*	Diff	Low *U*	High *U*	Diff
Parabolic coefficient	0.37	0.34	0.39	0.05	0.40	0.50	0.10	0.35	0.45	0.10
Height/Radius	0.58	0.27	0.57	0.30	0.25	0.68	0.43	0.05	0.72	0.67
Radius (µm)	0.74	0.44	0.55	0.11	0.50	0.70	0.20	0.14	0.76	0.62
Height (µm)	0.82	0.34	0.70	0.36	0.33	0.81	0.48	-0.19	0.89	1.08
Volume (million µm^3)	0.84	0.46	0.64	0.17	0.51	0.77	0.26	-0.05	0.84	0.89
Surface area (µm^2)	0.86	0.44	0.65	0.21	0.48	0.79	0.31	-0.08	0.86	0.94
Area (µm^2)	0.86	0.42	0.67	0.25	0.45	0.80	0.35	-0.13	0.87	1.00
Arc length (µm)	0.86	0.38	0.69	0.31	0.39	0.81	0.42	-0.15	0.89	1.04
Average across 8 traits	0.74	0.39	0.61	0.22	0.41	0.73	0.32	-0.01	0.79	0.79

Table S4. Comparison of prediction accuracy for forward and reverse prediction for all traits. The 369-accession training set (T369) and the 488-accession validation set (V488) were phenotyped in different experiments in 2013 and 2016, respectively. The forward prediction uses the T369 to train the model and predict V488. The reverse prediction uses the subset of materials in the V488 (244 accession high-*U* set or 244 accession low-*U* set) to train the model and predict the T369. *U*, upper bound for reliability.

Trait	Forward Prediction	Reverse Prediction	
	T369 → V488	High*U*244 → T369	Low*U*244 → T369
Parabolic coefficient	0.37	0.29	0.20
Height/Radius	0.45	0.44	0.22
Radius (µm)	0.51	0.53	0.30
Height (µm)	0.57	0.61	0.21
Volume (million µm^3)	0.56	0.62	0.34
Surface area (µm^2)	0.56	0.63	0.29
Area (µm^2)	0.57	0.63	0.27
Arc length (µm)	0.57	0.62	0.22
Average across 8 traits	0.52	0.55	0.26
